# Wastewater Metagenomic Reanalysis of Antibiotic Resistance Genes in Public Datasets from Türkiye (Ankara and Hatay)

**DOI:** 10.3390/antibiotics15080795

**Published:** 2026-08-17

**Authors:** Halil Kurt

**Affiliations:** Medical Biology Department, Hamidiye International School of Medicine, University of Health Sciences, Istanbul 34668, Türkiye; halil.kurt@sbu.edu.tr

**Keywords:** wastewater-based epidemiology, Türkiye, high quality metagenome-assembled genomes (HQ-MAGs), antibiotic resistance genes, resistome, antimicrobial resistance

## Abstract

**Background/Objectives**: Antimicrobial resistance in microbial communities is a global health concern that leads to millions of deaths each year. Many bacterial pathogens have resistance to multiple antibiotics. Domestic wastewater treatment facilities are reservoirs for antibiotic-resistant bacteria and resistance genes. Wastewater-based epidemiology surveillance is crucial for monitoring antibiotic resistance genes (ARGs). Türkiye has one of the highest levels of antibiotic resistance with a lack of research on resistomes. This study is a focused reanalysis of publicly available wastewater metagenomes from Türkiye, comparing them to global and other country’s results. **Methods**: Ten metagenomic data of wastewater treatment from Türkiye were downloaded from NCBI-SRA database. Metagenome assemblies were performed and high-quality metagenome-assembled genomes (HQ-MAGs) were included in the study. Taxonomic annotations and antibiotic resistance profiles were identified in both the metagenome assemblies and HQ-MAGs. **Results**: A total of 401 different ARGs in 25 antibiotic classes have been identified, including *Mcr* (including *mcr-1*, *mcr-2*, *mcr-3* and *mcr-5* variants) and *optrA*. The *vanR* two-component regulatory system genes for controlling vancomycin antibiotic resistance were one of the most dominant along with other vancomycin resistance genes such as *vanA* and *vanB*. A total of 115 HQ-MAGs were obtained with at least eight ARGs. The HQ-MAG with the highest number of resistance genes (58) was found to belong to *E. coli*. The most frequently encountered resistance genes in HQ-MAGs were the multidrug ABC transporter, *vanR*, *bacA* and *patA* which confer resistance to multidrug, glycopeptide, bacitracin and fluoroquinolone antibiotic groups, respectively. **Conclusions**: To effectively address the problems of antibiotic resistance outbreaks, comparable AMR surveillance at national and global levels is required for the identification and prioritization of ARGs and resistance genes. This is the first report conducted in Türkiye.

## 1. Introduction

Antimicrobial resistance in microbial communities, particularly to antibiotics, has emerged as a global health concern. Moreover, millions of people die each year due to infections caused by antibiotic-resistant bacteria [1]. A significant proportion of bacterial pathogens, notably ESKAPEE pathogens (*Enterococcus faecium*, *Staphylococcus aureus*, *Klebsiella pneumoniae*, *Acinetobacter baumannii*, *Pseudomonas aeruginosa*, *Enterobacter* spp., and *Escherichia coli*), have demonstrated resistance to multiple antibiotics, thereby complicating the treatment of diseases caused by these pathogens [2]. Consequently, the World Health Organization (WHO) has included these pathogens on its list of bacteria requiring the urgent development of new antibiotics [3]. Excessive utilization of antibiotics in agriculture, animal husbandry, and human medicine exerts selective pressure on microorganisms, thereby contributing to the global proliferation of antimicrobial-resistant bacteria and antibiotic resistance genes in all environmental sections, including soil, air, and water [4]. The increased presence of antibiotic resistance genes in nature, in conjunction with lateral gene transfer, which enables non-resistant bacteria to become resistant, further exacerbates the problem.

Wastewater treatment facilities are engineered to collect and treat domestic and industrial wastewater using microbial processes. Domestic wastewater, particularly hospital wastewater, has been found to contain elevated levels of pathogenic microorganisms and their associated antimicrobial resistance genes. These contaminants are transferred to wastewater treatment facilities, where they pose a significant challenge to the effective removal of pathogens. Consequently, wastewater treatment facilities are regarded as reservoirs for antibiotic-resistant bacteria and resistance genes, which are gathered, harbored, and disseminated into the environment [5]. Wastewater surveillance is imperative for the monitoring of antibiotic resistance and resistant organisms within the community [6]. Resistome refers to the assortment of antibiotic resistance genes (ARGs) harbored by all microorganisms in a particular ecological environment [7]. Consequently, metagenomic sequencing studies of wastewater samples are being conducted to identify all resistance genes, and these resistome studies are quite popular [8,9,10]. Indeed, resistome studies are being conducted on wastewater samples on a global scale [11,12,13].

To the best of our knowledge, there has been a lack of comprehensive research focusing on the resistome profile of wastewater treatment plants within Türkiye. In a resistome study conducted on a global scale, samples were also taken from Türkiye; however, they were mentioned very briefly in the article. Consequently, despite the collection of samples from wastewater treatment facilities within Türkiye, the comprehensive resistome profiles of these samples remain unreported. Our hypothesis was that resistome profile of wastewater treatment plants within Türkiye are similar to global counterparts.

The aim of this study is to extract resistome profiles using advanced bioinformatic analyses of metagenomic sequencing results from 10 wastewater treatment plant samples from Türkiye and compare them with global and other countries.

## 2. Results

The process of metagenomic sequencing involves the analysis of genetic material from a microbial community, encompassing the genetic composition of its constituent organisms. Metagenomic sequences of wastewater treatment plant samples collected from Türkiye (Ankara and Hatay) were retrieved from the NCBI SRA database and subsequently analyzed. The samples contained an average of 29 million reads. A total of 67% of these reads were successfully classified with Kraken 2 suite. The mean proportion of classified reads that belonged to bacteria was 99% with a standard deviation of 0.94%, while the mean proportion of classified reads that belonged to viruses was 0.27% with a standard deviation of 0.38%, as shown in Table S2. The prevalence of viruses was found to vary substantially across different samples. Specifically, ERR4678594 exhibited the highest percentage of viruses (1.138%), while ERR2607547 and ERR1726009 showed the lowest percentage (0.04%).

### 2.1. Shotgun Metagenome: Metagenome Analysis Reveals Diverse Microbial Communities

**Figure 1:**
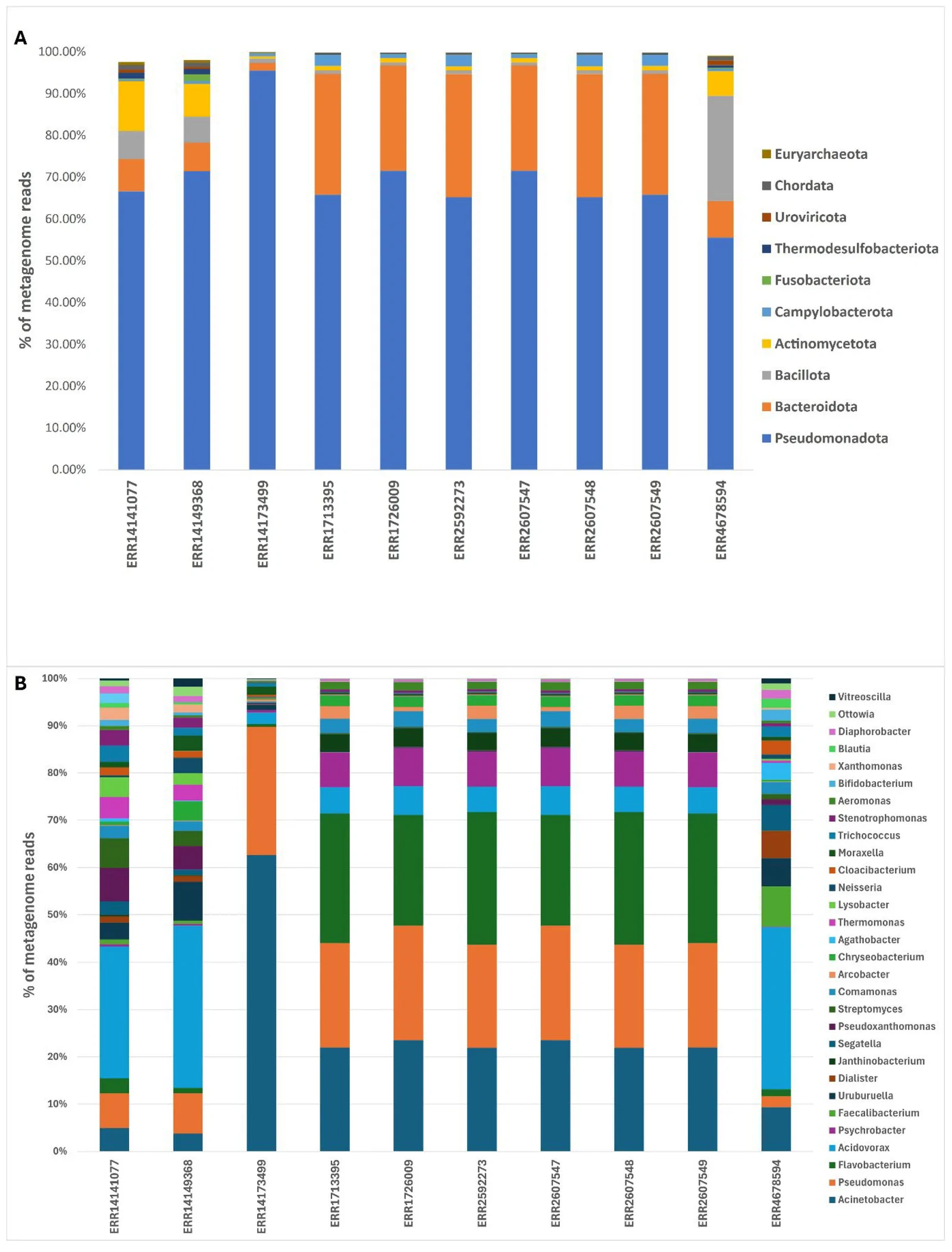
Composition of bacterial community in wastewater treatment systems. (**A**) Relative abundance of dominant (top 10) bacterial phylum. (**B**) Relative abundance of dominant (higher than 1%) bacterial genus.

The bacteria present in wastewater treatment systems that carry antibiotic resistance genes serve as reservoirs for ARGs and are responsible for the dissemination of these genes into receiving environments [14]. A comprehensive analysis of metagenomic data has led to the identification of 337 distinct species with an abundance greater than 0.01% across 31 distinct bacterial phyla from wastewater samples obtained within the provinces of Ankara and Hatay. Pseudomonadota, Bacteroidota, and Bacillota were identified as the predominant taxa at the phylum level in the samples examined (Figure 1A). It has been previously demonstrated through genomic analyses that species belonging to Pseudomonadota and Bacteroidota harbor a large number of antibiotic resistance genes [15].

Following shotgun metagenome analysis, a total of 30 genera with a prevalence of more than 1% were detected in all samples (Figure 1B). The Acinetobacter, Pseudomonas, Flavobacterium and Acidovorax are the dominant microorganisms detected in our samples. While the genera Acinetobacter and Pseudomonas are found in the environment, as opportunistic pathogens that colonize the human intestine, the genera Flavobacterium and Acidovorax are also present in activated sludge samples. The Flavobacterium genus is among the primary microorganisms encountered in wastewater treatment plants due to their ability to form flocs [16]. Acidovorax species are capable of denitrification and play a role in wastewater treatment process [17]. Although Acinetobacter, Pseudomonas, and Arcobacter are commonly found in wastewater treatment systems, there are also pathogenic species belonging to these genera. Since this study consists of MAGs by its very nature, verification at the culture or strain level is not feasible.

### 2.2. Multiple Antimicrobial Resistance Gene Classes Are Highly Prevalent in Ankara and Hatay Wastewater Treatment Samples

The composition of the AMR class has demonstrated regional characteristics at the global level. For instance, Europe and North America are geographically proximate, while Africa and Asia are located in different clusters [12]. According to previous studies, Türkiye is clustered with the European and North American countries [12,13].

**Figure 2:**
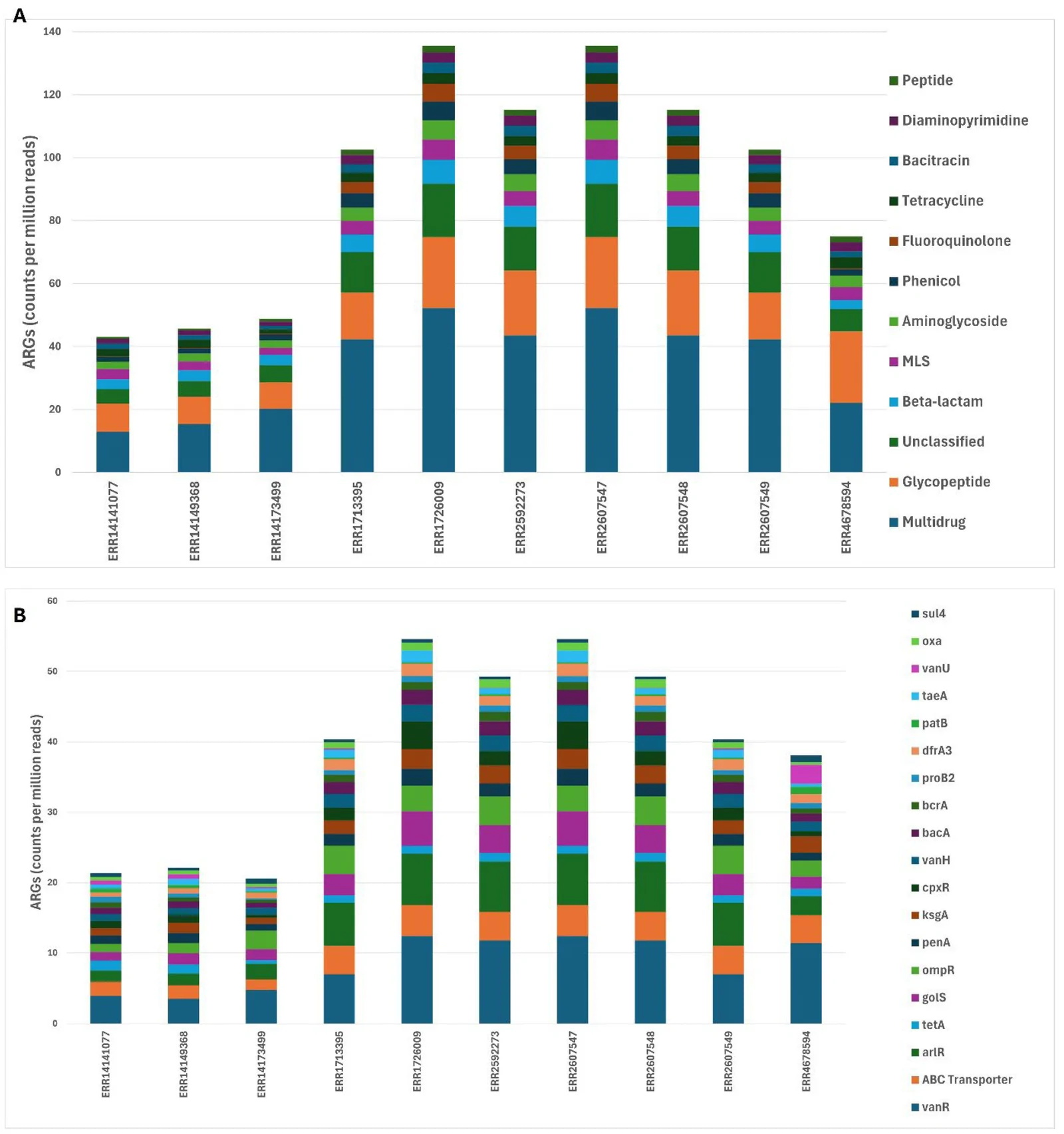
Quantitative overview of ARG occurrence in Ankara and Hatay wastewater treatment samples. (**A**) The relative abundance of 12 most common ARG classes. (**B**) The relative abundance of top 19 ARGs.

A total of 401 different ARGs belonging to 25 antibiotic classes were identified, including clinically important genes such as *mcr* (including *mcr-1*, *mcr-2*, *mcr-3* and *mcr-5* variants), and *optrA*. It is noteworthy that distinct ARGs may exhibit varied degrees of resistance to the same class of antibiotics. ARGs encoding resistance toward multidrug, Glycopeptide, Beta-lactam, MLS and Aminoglycoside were the most abundant (Figure 2A).

ARGs which are detected in all the samples are defined as core ARGs [18]. A total of 129 core ARGs were detected in our samples, and they were grouped into 13 antibiotic classes. Forty-six of them belong to the multidrug class of antibiotics, while 17 and 12 ARGs confer resistance to the MLS and glycopeptide classes of antibiotics, respectively. Genes involved in resistance to aminoglycoside, beta-lactam, and phenicol antibiotics were also found among the core ARGs. Similarly, multidrug, bacitracin, and tetracycline were the most frequently observed core ARG classes in wastewater samples from Slovakia and Taiwan [18].

Examining core resistance genes revealed that the *vanR* gene, which could cause glycopeptide resistance including vancomycin as a two-component regulatory system gene, was one of the most frequently encountered genes (Figure 2B). The multidrug ABC transporter gene, which causes multidrug resistance, was the second most frequently encountered gene. The high level of detection of these genes in all samples and their discharge into aquatic environments is a significant public health concern. A total of 557 different antibiotic resistance genes (ARGs) were detected in samples taken from the 747 urban wastewater treatment plant, 13 of which were present in all samples as core ARGs [12]. Similarly, we detected the *tetA* gene as well as the *tetB* and *tetR* genes, which are associated with tetracycline resistance, in all our samples.

### 2.3. Metagenome-Assembled Genomes: High Quality and Taxonomically Diverse MAGs Are Recovered

**Figure 3:**
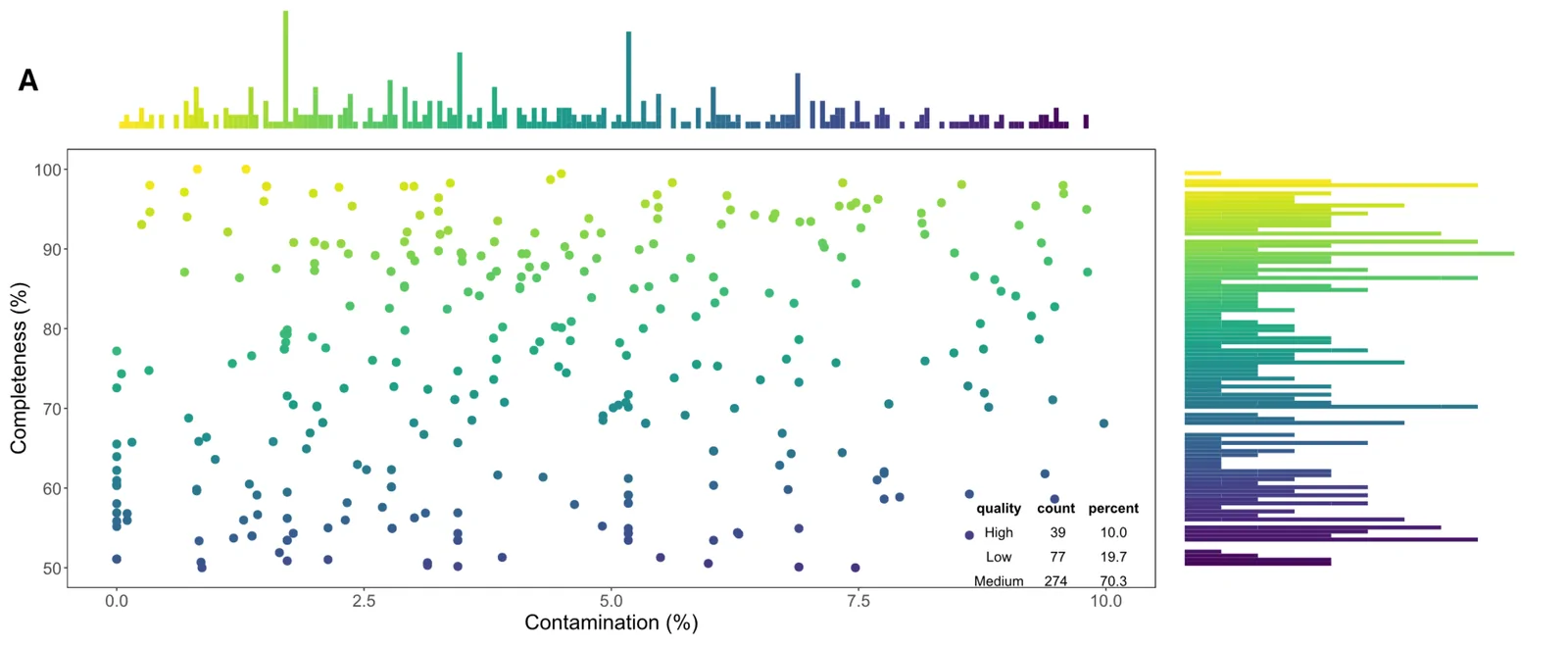

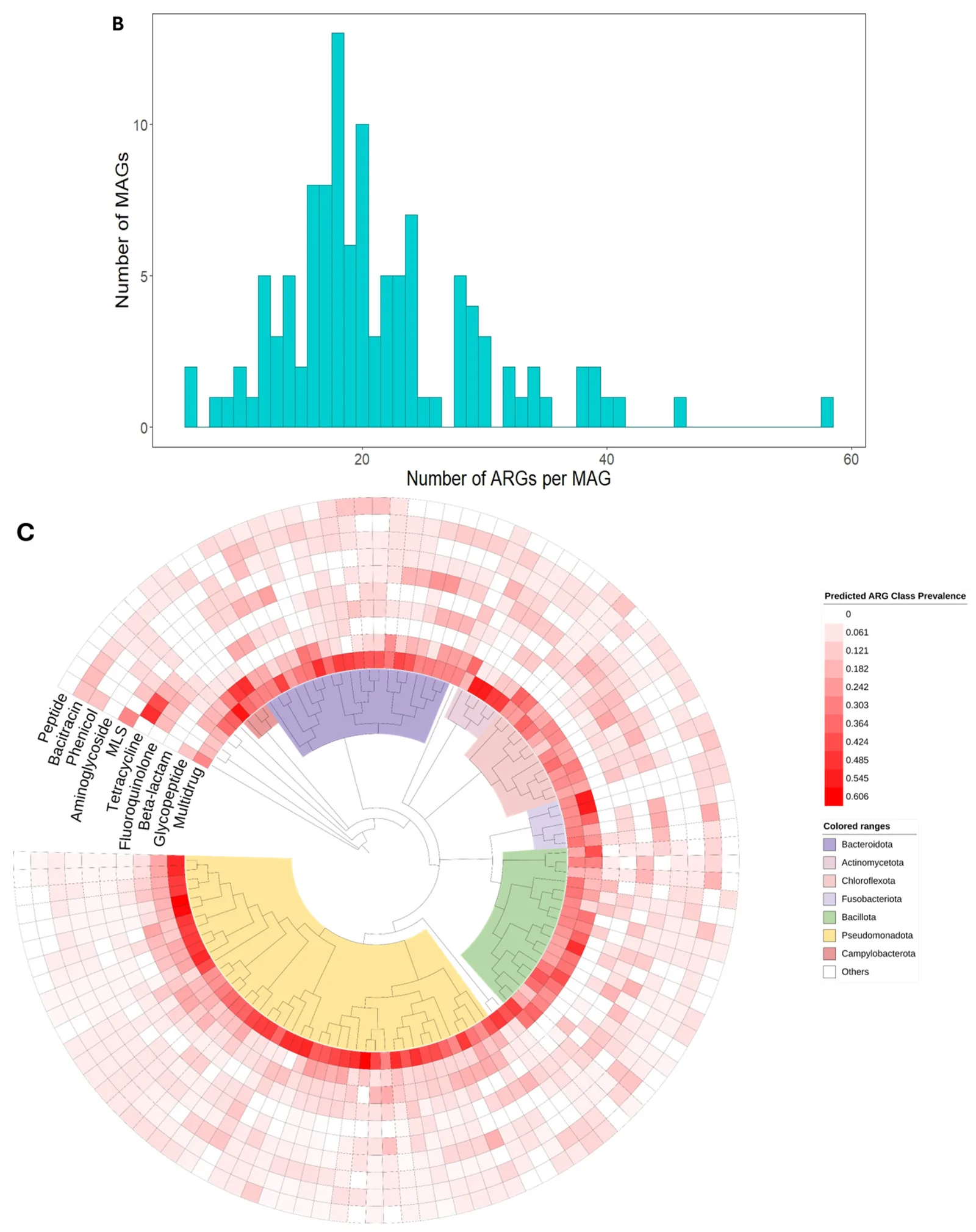
MAG quality and number of ARGs. (**A**) A scatter plot showing the contamination and completeness levels of the 390 MAGs. The two histograms show the distribution of contamination and completeness among the MAGs. For only (**A**), bins with ≥90% completeness and less than 5% contamination were considered high-quality MAGs while bins with completeness between 90% and %70 and with less than 10% contamination were considered medium-quality MAGs. Bins with ≤70% completeness and less than 10% contamination were considered low-quality MAGs and colored accordingly. (**B**) A histogram showing the number of ARGs found in each of the 115 MAGs. (**C**) A phylogenetic tree of MAGs based on their GTDB-tk taxonomy. Tree branches are colored based on their phyla. The surrounding heatmap shows per cluster the prevalence of ARGs belonging to the 10 most prevalent ARG classes in the MAGs.

**Figure 4:**
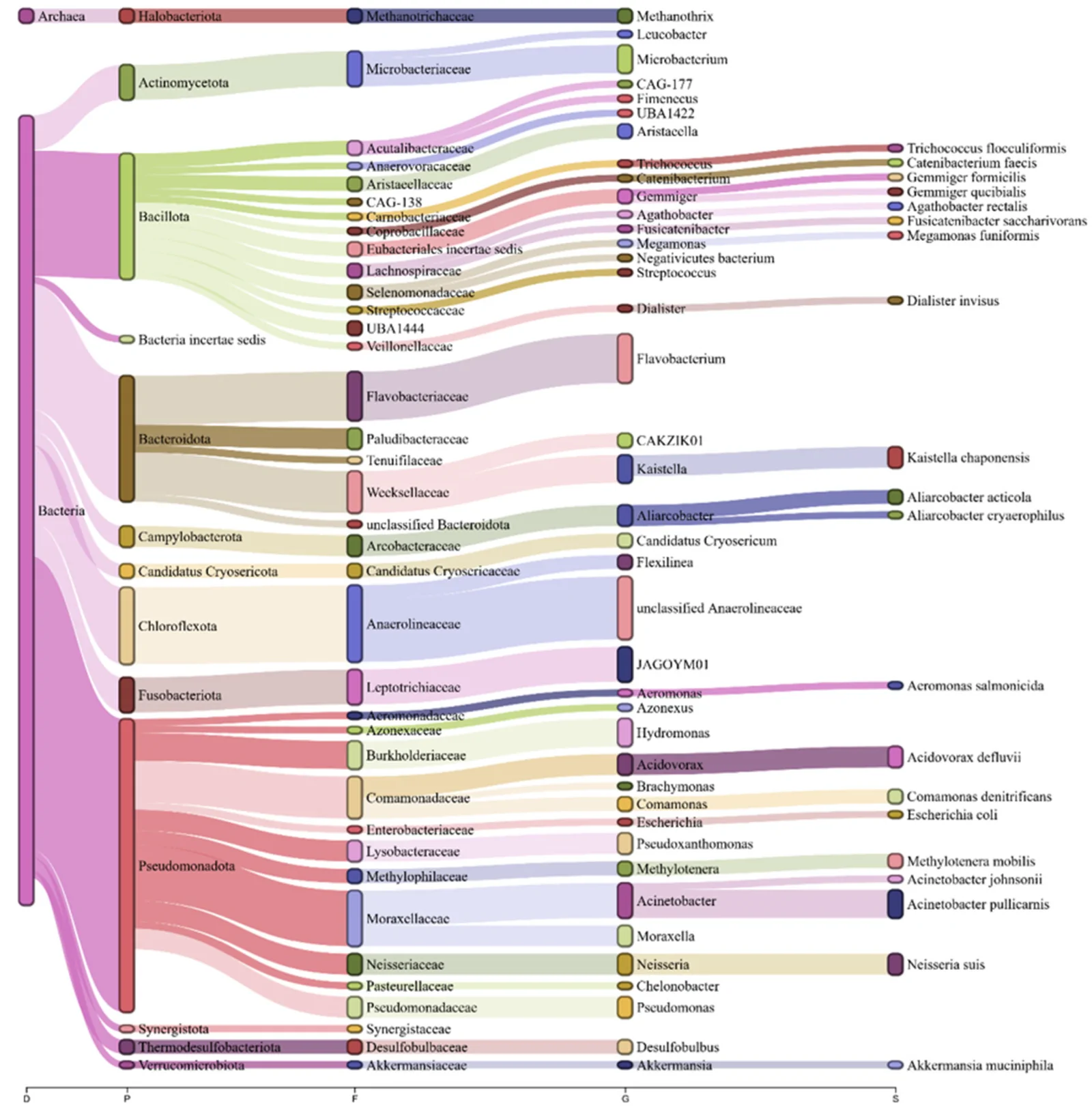
A Sankey diagram illustrating the abundance and taxonomic relationships of the 115 HQ-MAGs identified in our study. The columns represent the taxonomic levels: Domain, Phylum, Family, Genus, and Species. The strip widths are proportional to abundance. The connecting lines between the strips indicate the relationships among the taxonomic levels.

MAGs were generated from metagenome sequencing data obtained from wastewater treatment plants in Ankara and Hatay. A total of 390 MAGs (completeness ≥ 50%, contamination ≤ 10%, Figure 3A) were obtained. Of the 390 MAGs recovered, 10% were of high quality, while 70% were of medium quality (Figure 3A). A total of 115 high-quality MAGs (completeness ≥ 85%, contamination ≤ 5%) were obtained and selected for downstream analysis (Table S3). Most of the recovered MAGs were from bacteria, while two MAGs belonged to archaea. According to GTDB-Tk assignments, Pseudomonadota (44 MAGs) was the most abundant phylum, followed by Bacteroidota (20 MAGs) and Bacillota (18 MAGs) (Figure 3C). MAGs with taxonomy at the species level accounted for approximately 24%. Comamonas, Neisseria and Pseudomonas were the most frequently detected genera in MAGs (Figure 4).

### 2.4. Multiple Antimicrobial Resistance Gene Classes Are Highly Prevalent in All Identified MAGs

**Figure 5:**
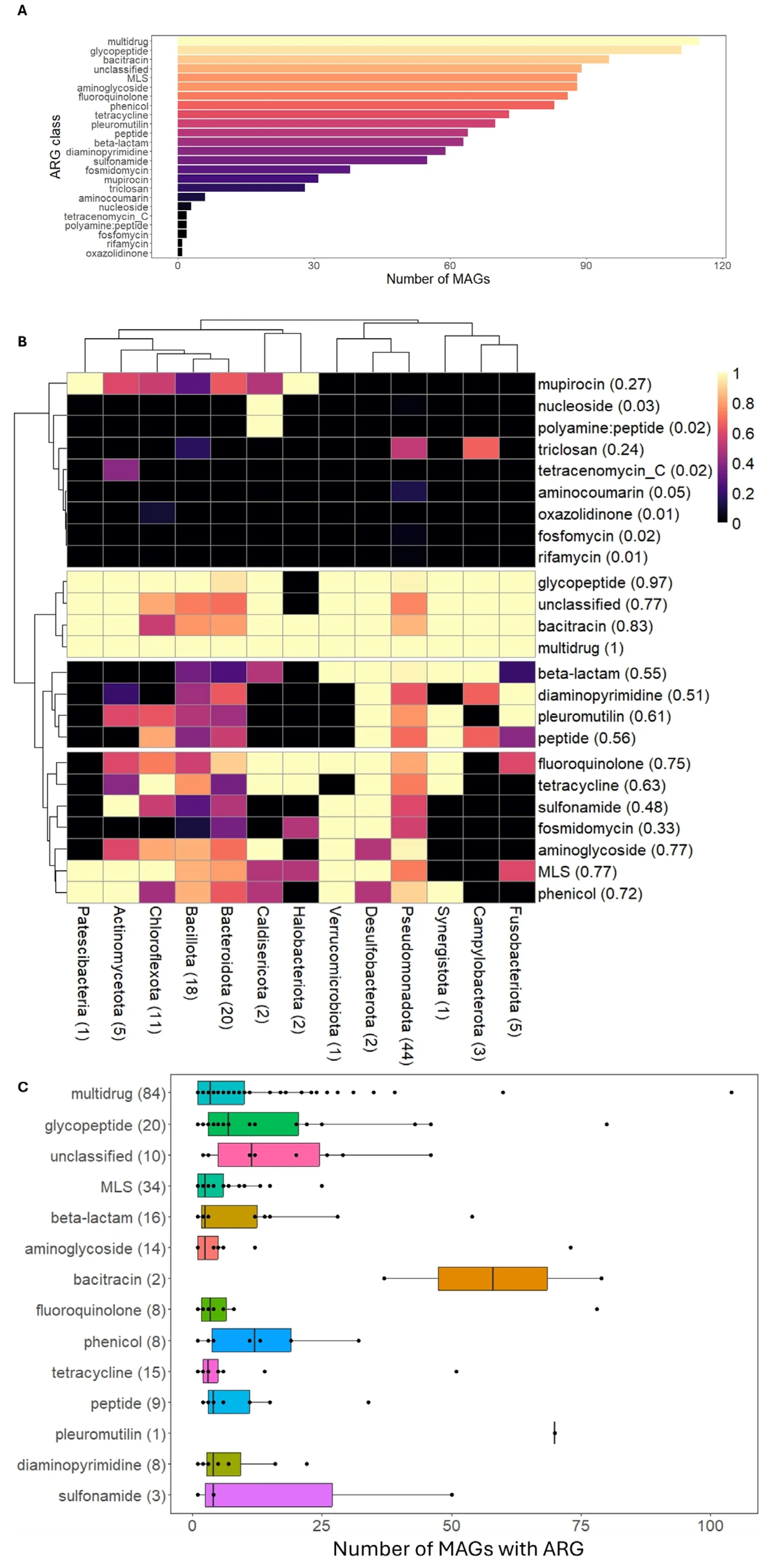
Prevalence of antimicrobial resistance gene classes in Ankara and Hatay wastewater treatment samples. (**A**) A bar plot showing the number of MAGs that contain ARGs belonging to each ARG class. Colors refer to the prevalence of each ARG class in all MAGs. (**B**) A heatmap showing the prevalence of ARG classes in the urban taxonomic phylum level. Shown are the number of MAGs that belong to each phylum. In gray within the legend, we show the ranges of our defined prevalence categories. (**C**) Boxplots showing the number of MAGs that contain each ARG within the ARG classes. In parentheses are the number of ARGs within the ARG class.

ARGs in each genome were identified using the deepARG program. All MAGs contained at least eight ARGs. ERR4678594_bin11 (*Escherichia coli*) carried the highest number of resistance genes, with 58 ARGs. The average number of ARGs being carried was determined to be 21.42 ± 8.51 (Figure 5A). Multidrug-related resistance genes were detected in all MAGs. Glycopeptide and Bacitracin class resistance genes were also found to be highly prevalent among MAGs (Figure 5A). MLS, aminoglycoside, fluoroquinolone, and phenicol class resistance genes were detected in at least 80% of MAGs.

The prevalence of all ARG classes among the MAGs was calculated (Figure 5B). Cases with a prevalence greater than 0.75 were defined as prevalent. Those with a prevalence between 0.5 and ≤0.75 were classified as common. Cases with a prevalence between 0.25 and ≤0.5 were labeled as rare, and those with a prevalence less than 0.25 were considered infrequent. The analysis revealed four distinct clusters. The most observed resistance class was multidrug, glycopeptide, and bacitracin resistance genes.

The following top five genes were identified in MAGs: multidrug ABC transporter (104 MAGs), *vanR* (80 MAGs), *bacA* (79 MAGs), *patA* (78 MAGs) and *taeA* (70 MAGs). These findings indicate resistance to multidrug, glycopeptide, bacitracin, fluoroquinolone and Pleuromutilin antibiotic groups, respectively (Table 1). Multidrug resistance was identified as the most prevalent type of resistance in the samples examined. A total of 84 different resistance genes were detected in this class, along with the most frequently encountered multidrug ABC transporter (Figure 5C). The multidrug ABC transporter functions as an antibiotic efflux pump, and its presence was detected in 104 MAGs. The *vanR* gene, a two-component regulatory system gene for controlling vancomycin antibiotic resistance, was identified in a total of 80 MAGs, thereby conferring resistance to glycopeptide antibiotics (see Table 1 for details). In addition, the *bacA* gene, the third most prevalent gene identified in 79 MAGs, is implicated in the development of resistance to bacitracin group antibiotics through chemical alterations. The *PatA* gene serves as an antibiotic efflux pump which is responsible for Fluoroquinolone resistance [19,20].

## 3. Discussion

Metagenome analysis reveals diverse microbial communities with a total of 30 genera (>1%) in all samples (Figure 1B). The Acinetobacter, Pseudomonas, Flavobacterium and Acidovorax were dominant genera. Acinetobacter species, particularly *A. baumannii*, are one of the most common causative agents of nosocomial and community-acquired infections [21,22]. Acinetobacter species are intrinsically resistant to many antibiotics. They can also rapidly develop resistance to new antibiotics via genetic elements such as plasmids, transposons, and integrons [23]. *Acinetobacter* spp. strains resistant to almost all antibiotics except colistin and tigecycline have increased rapidly in recent years. Türkiye, along with Italy, Spain, and Greece, is one of the countries in Europe where multidrug-resistant Acinetobacter species are most frequently encountered [24]. A rapid increase in multidrug-resistant *A. baumannii* strains has been observed in Türkiye over the past decade because of the selective pressure of increasing antibiotic use [25]. Therefore, it is important to monitor increasing antimicrobial resistance levels in Acinetobacter species through wastewater-based resistome studies at the community level.

Pseudomonas, considered one of the most diverse and widespread groups, are essential members of the microbial community in wastewater treatment processes. Pseodomanas contain human and animal pathogenic species such as *P. aeruginosa*, *P. fluorescens*, *P. putida*, *P. stutzeri*, and *P. anguilliseptica* [26]. Pseudomonas can develop resistance to many antibiotics because of its genomic plasticity [27]. Over 90% of Pseudomonas species isolated from wastewater have shown resistance to at least one antibiotic. In addition, multidrug resistance has been observed in *P. putida*, *P. aeruginosa*, *P. fluorescens*, *P. pseudoalcaligenes*, and *P. protegens* isolates [28].

*Flavobacterium* spp. isolated from wastewater treatment plants have been found to harbor resistance genes against numerous antibiotics, including Aztreonam, Cefadroxil, Ceftazidime, Metronidazole, Trimethoprim, and Trimethoprim-sulfamethoxazole [29]. Thus, wastewater treatment plants harbor many antibiotic-resistant bacteria, making it important to monitor resistome profiles for public health reasons.

A total of 401 different ARGs, including mcr (including mcr-1, mcr-2, mcr-3 and mcr-5 variants) *bacA*, *patA* and *taeA* have been found. ARGs may exhibit varying degrees of resistance to the same class of antibiotics. ARGs against multidrug, glycopeptide, beta-lactam, MLS and aminoglycoside antibiotics were most abundant. Similar to our study, Beta−Lactam, glycopeptide, as well as Aminoglycoside and Sulfonamide resistance were most common in other wastewater-based resistome studies [11,12,13].

Genes encoding major facilitator superfamily (MFS) and ABC transporters, two efflux-system classes, were highly prevalent. *ArlR* is a response regulator that activates the expression of the *norA* promoter belonging to the MFS transporter family [30].

Vancomycin, a glycopeptide antibiotic, functions by binding to the terminal D-Ala-D-Ala peptidoglycan precursors in the bacterial cell wall, thereby impeding cell wall synthesis. The antibiotic resistance genes *vanR*/*vanS* act as two-component regulatory system when detecting vancomycin and turn on the resistance genes. *vanH*, *vanA/B*, and *vanX* modify the terminal D-Ala-D-Ala peptidoglycan structure, thereby conferring resistance to vancomycin [31]. A thorough analysis of the samples reveals that the *vanR* gene is the most prevalent gene which regulates vancomycin resistance.

When the ARGs were grouped by drug class, the most frequently encountered ARGs in the samples were those belonging to the following categories: multidrug, glycopeptide, beta-lactam, MLS, and aminoglycosides. The relative abundance of ARGs encoding drug classes was consistent across samples, but the ARG load in samples from Hatay was lower than in those from Ankara. It has been determined that temperature has significant effects on the abundance and distribution of ARGs due to the increased removal efficiency of ARGs as a result of temperature [32]. A positive correlation has been identified between urban population size and the abundance of ARGs [18]. The increased usage of antibiotics and elevated wastewater levels in densely populated urban areas may result in an amplified abundance and distribution of antibiotic resistance genes (ARGs) in bacterial populations [33]. This explains our observation to some extent. However, a more detailed and comprehensive study should be conducted.

Surveys of the global resistome have indicated that resistome profiles exhibit local clustering and demonstrate regional stability [12]. Analyses at the regional level are also essential for understanding resistome dynamics in urban wastewater treatment plants, as they inform the development of future management strategies [18] (Table 2). Consequently, our study has delineated the local resistome profile that is unique to our nation, thereby establishing a foundational framework for subsequent research endeavors.

In recent years, a significant number of metagenome-assembled genomes have been obtained from soil [34], human gut [35], and wastewater treatment [36] samples based on culture-independent metagenome sequencing. The MAGs approach has been used to elucidate their genomic potential and resistome profiles of microbial communities [36,37]. In our study, 115 high-quality MAGs affiliated to Bacteria (113) and Archaea (2) were obtained. Pseudomonadota was the most abundant phylum according to GTDB-Tk assignments. The study of urban wastewater treatment plant samples yielded 4281 microbial artificial genomes (MAGs), predominantly classified within the Actinobacteria, Bacteroidota, Firmicutes, and Proteobacteria phyla [36]. In the present study, MAGs belong to Pseudomonadota, Bacteroidota, and Bacillota phyla were dominant in concordance to microbial diversity of wastewater treatment studies [38].

Approximately 24% of MAGs had taxonomy at the species level, and most frequently detected genera were Comamonas, Neisseria and Pseudomonas. Metagenome-assembled genomes indicate that antimicrobial resistance genes are highly prevalent among urban bacteria and multidrug and glycopeptide resistances are ubiquitous in most taxa. This approach is particularly useful in elucidating the relationship between resistance genes and their host bacteria.

**Table 2:** Summary and main conclusion of wastewater-based resistome studies.

Region Country	Sample Type	Number of Samples	Methods	ARG Database/Software	Mobilome Analysis	MAG Analysis	Dominant ARG Classes	Main Conclusion	Reference
Portugal, Spain, Ireland, Cyprus, Germany, Finland, and Norway	wastewater	12	QPCR	QPCR array	Yes	No	beta-Lactam, Tetracycline, Aminoglycoside, MDR, MLS	Need to implement regular surveillance and control measures	[10]
Global (79 sites in 60 countries)	urban sewage	79	Metagenome sequencing	ResFinder	No	No	Phenicol, Sulphonamide, Tetracycline, Aminoglycoside, Beta−Lactam	Economically feasible approach for continuous global surveillance and prediction of AMR	[11]
243 cities in 101 countries	sewage	757	Metagenome sequencing	ResFinder	Yes	No	Aminoglycoside, Beta−lactam, Fluoroquinolone, Folate pathway antagonist, Fosfomycin	Certain geographies are more prone to transmission events and should receive additional attention	[12]
Global, 23 countries	activated sludge	226	Metagenome sequencing	CARD	Yes	Yes	Beta−lactam, Glycopeptide, Tetracycline, Aminoglycoside, Sulfonamide	Resistome variations appear to be driven by a complex combination of stochastic processes and deterministic abiotic factors.	[13]
Agadir city, Morocco	wastewater treatment	6	QPCR	QPCR primers	No	No	aminoglycoside, macrolide, lincosamide, beta-lactamase, chloramphenicol, sulfonamide	Additional treatment is necessary to eliminate ARGs, ensuring that the water can be safely utilized without posing health risks	[39]
Slovakia and Taiwan	urban wastewater	12	Metagenome sequencing and QPCR	CARD	No	No	Multidrug, MLS, beta-lactam, Bacitracin, polymicin	Optimizing treatment processes and reducing the dissemination of antibiotic resistance and pathogenicity genes for safeguarding public health	[18]
Virginia, United States	domestic wastewater	22	Metagenome sequencing and QPCR	CARD	No	No	Aminocoumarin, Aminoglycoside, Beta-lactam, MLS, Peptide	Effluent is a beneficial monitoring point with relevance both to the local clinical condition and for assessing efficacy of wastewater treatment in reducing risk of disseminating antibiotic resistance.	[6]

Since our study used MAGs instead of purified cultures, the identified ARGs, while providing strong evidence of resistance, do not provide phenotypic evidence as tests conducted on purified cultures do. However, wastewater-based MAG studies are a very robust method, as they enable the rapid and cost-effective screening of antimicrobial resistance at the resistome level.

Despite obtaining high-quality MAGs, bias is introduced in the interpretation of results compared to clinical isolates due to the contamination and completeness of MAGs. Since the amount and classes of antibiotics prescribed by doctors at the time and location of sample collection are unknown, it is not possible to establish a correlation between the resistome profiles and prescription of antibiotics.

## 4. Materials and Methods

### 4.1. Metagenome Selection

We extracted a total of 10 metagenomic samples belonging to Türkiye from the Global surveillance of antimicrobial resistance [12] and global Water Microbiome Consortium [13]. Metagenomics reads belonged to BioProject numbers of PRJEB13831, PRJEB27054, PRJEB40798, PRJEB40816, and PRJEB84064 (Table S1). Readings were downloaded from the NCBI SRA database (https://www.ncbi.nlm.nih.gov/sra/ accessed on 20 June 2025).

### 4.2. Pre-Processing and Library Assembly

Quality filtering of samples and removal of primers and adapters were performed using Trimmomatic v0.38 [40] with parameters of ILLUMINACLIP:TruSeq3-PE.fa:2:30:10 LEADING:3 TRAILING:3 SLIDINGWINDOW:4:15 MINLEN:36. The remaining clean reads were assembled using the megahit assembler with the meta-sensitive parameter [41]. The metaWRAP::Binning module [42] was used with the programs MaxBin2 (v2.2.6) [43], CONCOCT (v1.0.0) [44] and metaBAT2 (v2.12.1) [45] to obtain individual bins. We performed a quality check of the obtained bins with CheckM (v1.0.12) [46]. The bins were refined using the MetaWRAP::Bin_refinement [42] module. Bins with ≥70% completeness and less than 5% contamination were considered high-quality MAGs and selected for further analysis as HQ-MAGs. For only Figure 3A, bins with ≥90% completeness and less than 5% contamination were considered high, while bins completeness between 90% and %70 and with less than 10% contamination were considered medium. Bins with ≤70% completeness and less than 10% contamination were considered low-quality MAGs.

### 4.3. Taxonomy Classification and Antimicrobial Gene Annotation

Taxonomic annotation of selected genomes was done with the GTDB-Tk v2.3.2 [47]. Phylogenetic trees were constructed using GTDB-tk taxonomic classifications. iTOLs was used to visualize the constructed tree and generate a heatmap of antibiotic groups (https://itol.embl.de/ accessed on 17 August 2025) Kraken suite has been used for taxonomic annotations in assembled reads. Suite contained Kraken 2 [48] with Standard r220 database and bracken [49] for taxonomic annotation. The Pavian metagenomics data explorer was used in the comparative analysis of the samples [50]. DeepARG v1 [51], a deep learning-based program, was used to identify antibiotic resistance genes. DeepARG-SS model was used for metagenomic reads and MAGs with LS model, --min-prob 0.8, --arg-alignment-identity 30, and --arg-alignment-evalue 1 × 10^−10^ parameters. Database v2 was used for deepARG search. ARG and ARG class prevalences within MAGs were quantified based on gene presence/absence profiles in individual MAGs. For each MAG, prevalence represents the number of positive MAGs divided by the total number of MAGs in that cluster [36].

## 5. Conclusions

To effectively address antibiotic resistance challenges, comparable AMR surveillance at the national and global levels is necessary for the identification and prioritization of ARGs and resistance genes. While the investigation of resistomes in wastewater samples is imperative for elucidating antibiotic resistance genes (ARGs) and their resistance profiles, it is equally crucial to undertake more detailed and comprehensive studies on the distribution of these ARGs in nature and their possible transmission to pathogens.

## Figures and Tables

**Table 1:** Resistance mechanisms of the ARGs that were found in more than 50 MAGs.

ARG	ARG Class	Resistance Mechanism	Number of MAGs
Multidrug ABC transporter	Multidrug	Antibiotic efflux	104
*vanR*	Vancomycin	Two-component regulatory system	80
*bacA*	Bacitracin	Antibiotic target alteration (recycles undecaprenyl pyrophosphate during cell wall biosynthesis which confers resistance to bacitracin)	79
*patA*	Fluoroquinolone	Antibiotic efflux	78
*taeA*	Pleuromutilin	Antibiotic efflux	70
*penA*	Beta-lactam	Antibiotic target alteration	54
*tetA*	Tetracycline	Antibiotic efflux	51
*sul4*	Sulfonamide	Antibiotic target replacement	50

## Data Availability

Raw metagenomics reads used in current study belonged to BioProject numbers of PRJEB13831, PRJEB27054, PRJEB40798, PRJEB40816, PRJEB84064 were downloaded and accessible from the NCBI SRA database (https://www.ncbi.nlm.nih.gov/sra/ accessed on 20 June 2025). HQ-MAGs generated and/or analyzed during the current study are available in the zenodo repository, https://doi.org/10.5281/zenodo.20539081.

## Supplementary Materials

The following supporting information can be downloaded at: https://www.mdpi.com/article/10.3390/antibiotics15080795/s1, Table S1: Accession numbers of SRA metagenomics read; Table S2: Taxonomic diversity of metagenomics read; Table S3: Statistics and taxonomy of HQ-MAGs statistics; Table S4: DepARG results of all MAGs.

## Abbreviations

The following abbreviations are used in this manuscript:

ARGs	Antibiotic resistance genes
HQ-MAGs	High-quality metagenome-assembled genomes
AMR	Antimicrobial resistance
ESKAPEE	*Enterococcus faecium*, *Staphylococcus aureus*, *Klebsiella pneumoniae*, *Acinetobacter baumannii*, *Pseudomonas aeruginosa*, *Enterobacter* spp., and *Escherichia coli*
WHO	World Health Organization
NCBI SRA	National Center for Biotechnology Information Sequence Read Archive
